# Implementation strategies and outcome measures for advancing learning health systems: a mixed methods systematic review

**DOI:** 10.1186/s12961-023-01071-w

**Published:** 2023-11-27

**Authors:** Mari Somerville, Christine Cassidy, Janet A. Curran, Catie Johnson, Douglas Sinclair, Annette Elliott Rose

**Affiliations:** 1 IWK Health, Halifax, NS Canada; 2 Faculty of Health, School of Nursing, Dalhousie University, Halifax, NS Canada

## Abstract

**Background:**

Learning health systems strive to continuously integrate data and evidence into practice to improve patient outcomes and ensure value-based healthcare. While the LHS concept is gaining traction, the operationalization of LHSs is underexplored.

**Objective:**

To identify and synthesize the existing evidence on the implementation and evaluation of advancing learning health systems across international health care settings.

**Methods:**

A mixed methods systematic review was conducted. Six databases (CINAHL, Embase, Medline, PAIS, Scopus and Nursing at Allied Health Database) were searched up to July 2022 for terms related to learning health systems, implementation, and evaluation measures. Any study design, health care setting and population were considered for inclusion. No limitations were placed on language or date of publication. Two reviewers independently screened the titles, abstracts, and full texts of identified articles. Data were extracted and synthesized using a convergent integrated approach. Studies were critically appraised using relevant JBI critical appraisal checklists.

**Results:**

Thirty-five studies were included in the review. Most studies were conducted in the United States (*n* = 21) and published between 2019 and 2022 (*n* = 24). Digital data capture was the most common LHS characteristic reported across studies, while patient engagement, aligned governance and a culture of rapid learning and improvement were reported least often. We identified 33 unique strategies for implementing LHSs including: change record systems, conduct local consensus discussions and audit & provide feedback. A triangulation of quantitative and qualitative data revealed three integrated findings related to the implementation of LHSs: (1) The digital infrastructure of LHSs optimizes health service delivery; (2) LHSs have a positive impact on patient care and health outcomes; and (3) LHSs can influence health care providers and the health system.

**Conclusion:**

This paper provides a comprehensive overview of the implementation of LHSs in various healthcare settings. While this review identified key implementation strategies, potential outcome measures, and components of functioning LHSs, further research is needed to better understand the impact of LHSs on patient, provider and population outcomes, and health system costs. Health systems researchers should continue to apply the LHS concept in practice, with a stronger focus on evaluation.

**Supplementary Information:**

The online version contains supplementary material available at 10.1186/s12961-023-01071-w.

## Introduction

Learning health systems (LHSs) were first defined by the Institute of Medicine in 2007, as a system where “science, informatics, incentives, and culture are aligned for continuous improvement and innovation, with best practices seamlessly embedded in the delivery process and new knowledge captured as an integral by-product of the delivery experience” [1]. This approach to health system restructuring provides a promising opportunity to enhance value-based healthcare (VBHC). Value-based healthcare (VBHC) places patients at the forefront of health care services, while emphasizing quality of care over number of healthcare interactions [2, 3]. This approach also aims to reduce costs without sacrificing value [2, 4]. LHSs also place patients at the centre of the health system, with continuous learning from patient experience and outcome data cycling back into the system to improve care [5]. The LHS concept therefore aligns with the goals of VBHC [6]. There has been a global shift towards VBHC, and this concept is now recognized as a top health system priority [7, 8]. Despite the opportunity for LHSs to achieve VBHC across health systems, there are gaps in our understanding of how to operationalize LHSs.

Since its inception in 2007, the idea of LHSs has evolved to include several descriptions and features. Lavis et al*.* (2018) proposed seven characteristics reflective of a LHS: (1) engaged patients; (2) digital capture, linkage and timely sharing of relevant data; (3) timely production of research evidence; (4) appropriate decision supports; (5) aligned governance, financial and delivery arrangements; (6) culture of rapid learning and improvement; and (7) competencies for rapid learning and improvement [9]. In 2019, Menear and colleagues developed a LHS framework comprised of four key elements: (1) core values; (2) pillars and accelerators; (3) processes; and (4) outcomes [6]. The framework presents a structure in which health systems can work towards delivering more VBHC [6]. Another review by Zurynski and colleagues (2020) included over 200 LHSs papers and reported on the LHS terminology, frameworks, barriers and enablers of LHSs across the literature [10]. Studies in this scoping review used varying terms to describe LHSs, including learning health networks, rapid learning systems and learning healthcare systems [10]. Clearly, LHSs are gaining traction as a valuable model for healthcare organizations, and despite the varied terminology, the central focus on rapidly incorporating evidence into practice to enhance VBHC remains consistent.

While there is ample literature describing LHS characteristics, there is little information on how to put this model into practice. Effective implementation of LHSs has the potential to improve patient outcomes, reduce costs and enhance quality care [6]. So, without a proper understanding of LHS implementation, research and health system resources are lost. A narrative review of LHS frameworks by Allen et al., identified a roadmap to assist organizations in creating LHSs [11]. The authors presented a logic model with key inputs, outputs and outcomes, based on the core features of 17 LHS frameworks and models [11]. This study is a valuable resource to help health systems move towards a LHS model, however, this roadmap has not yet been applied to LHS-focused studies. Further, to date, no reviews have explored the types and outcomes of implementation strategies used by existing LHSs. As such, while the idea of LHSs is promising, it is still unclear how LHSs are operationalized across different health care organizations and countries. The scoping review by Zurynski et al*.* identified several functioning LHSs, but they did not describe how these LHSs were implemented or the outcomes of the implementation process [10]. Evidently, there is a need to unpack and synthesize the implementation process of LHSs across health care settings.

Implementation science is a field of research focused on methods and strategies to facilitate the uptake of evidence-based intervention and policies. Implementation strategies are techniques used to support the effective uptake of an intervention [12]. The Expert Recommendations for Implementing Change (ERIC) taxonomy includes a comprehensive list of 73 implementation strategies that were developed based on a review of the evidence and expert consultation [12]. Implementation scientists often apply these strategies in their research to ensure an intervention is delivered in a systematic, evidence-based way. Further, measures related to the implementation process can provide helpful information about whether an intervention led to meaningful change [13]. Tierney et al*.* established a list of 10 implementation measures specifically for evidence synthesis studies that provide additional insight as to the value of an intervention or research project [13]. While both the ERIC taxonomy and Tierney’s implementation measures have been applied to previous implementation research, they have not yet been applied to LHS-focused evidence synthesis work. With a clear gap in the evidence related to LHS implementation, there is an opportunity to understand how LHSs have been designed. Therefore, the aim of this study is to systematically synthesize the evidence on the implementation of LHSs across different health care organizations and countries. This aim will be achieved through the following objectives:

- Describe the LHS characteristics used across studies and health care organizations
- Identify the number and types of implementation strategies used to transition to a LHS
- Describe the LHS outcome measures applied across studies

## Methods

### Study design

A mixed methods systematic review (MMSR) was conducted following the Joanna Briggs Institute (JBI) methodological guidelines for MMSR [14]. A MMSR allows for the comprehensive overview of a broad research question or phenomenon of interest and may include evidence from qualitative, quantitative, and mixed methods study designs.

This review was registered in PROSPERO (CRD42022293348) and the protocol was previously published [15]. The Preferred Reporting in Systematic Reviews and Meta-Analyses (PRISMA) checklist was used to report the findings of this study [16].

### Inclusion criteria

Following JBI guidelines for MMSR [14], the PICo (Population; Phenomenon of Interest; Context) framework guided the question development and identification of inclusion criteria. This review aimed to answer the following research question: *How do healthcare organizations implement and evaluate the transformation of learning health systems?*

#### Population

Studies were considered for inclusion if they described a LHS, including rapid learning systems, rapid learning healthcare, learning healthcare systems, learning health systems or other similar LHS terms. Due to the inconsistency in reporting on LHSs, studies which described components of a LHS without using LHS terminology were excluded.

#### Phenomenon of Interest

This review included studies reporting on implementation strategies and/or outcome measures associated with the adaptation of LHSs. Implementation strategies include any procedure, approach, or method to implement, assess or evaluate the uptake of LHSs. The ERIC taxonomy of 73 strategies was used to identify implementation strategies reported across studies [12]. Any reported outcome measures were identified using Tierney et al.’s list of 10 implementation measures [13] to provide further insight into the value of the intervention and implementation process. Studies were further tagged as either provider, patient, population or healthcare cost-related outcomes, to reflect the quadruple aim of enhancing health systems [17, 18].

#### Context

Studies conducted in any health care setting were included. Health care settings may include hospitals, academic medical centres, primary care clinics, community health centres, practice-based networks or individual departments or clinics that provide health care to patients. Any country and size of healthcare organization were considered for inclusion. Non-healthcare settings, such as academic institutions, government, or non-government organizations where care is not directly provided to patients, were excluded.

#### Types of studies

Quantitative, qualitative, and mixed methods studies were considered for inclusion in this review. Additionally, descriptive papers reporting on the implementation of LHSs were included if they were peer reviewed. Grey literature sources such as policy reports, case studies or conference proceedings were included if they described the implementation strategies and/or outcome measures of LHSs. Protocol papers were excluded but forward citation searching was conducted to find any published studies stemming from the protocol. Similarly, reviews were excluded but the reference lists of identified reviews were manually searched for relevant papers.

### Search strategy

Six databases were searched up to July 2022, for key terms related to LHSs, implementation and health care, using a comprehensive search strategy developed by a research librarian trained in knowledge synthesis. The search strategy was peer reviewed (PRESSed) by an independent research librarian to validate the approach. The databases included CINAHL (EBSCOhost), Medline (Ovid), Embase (Elsevier), Nursing and Allied Health Database (ProQuest), PAIS (ProQuest) and Scopus (Elsevier). Boolean operators and MESH terms were used accordingly for each database. An example search strategy for CINAHL can be found in Additional file 1: Table S1. No restrictions were placed on date of publication or language. A grey literature search was conducted to identify additional relevant articles. This involved searching ProQuest’s Dissertations and Theses Global, a targeted search of the websites of three pre-identified relevant organizations, and a systematic Google search to identify relevant sources. The grey literature search strategy can be found in Additional file 1: Table S2 while a comprehensive description of the search strategy can be found in the published protocol paper [15]. Additional articles were retrieved through backward and forward citation searching of reference lists of included articles.

### Study selection

Identified articles were uploaded to the data management software, Covidence (Veritas Health Innovation, Melbourne, Australia), and duplicates were removed electronically. Two reviewers (MS and CJ) independently screened the titles, abstracts and full texts of identified articles based on the predetermined inclusion criteria. Any discrepancies in screening decisions were resolved through discussion by the reviewers, with an independent, third reviewer (CC), helping to reach consensus as needed. Studies were deemed eligible for inclusion if they reported on the implementation of LHSs, as outlined in the predetermined inclusion/exclusion criteria. One reviewer screened the first five pages of the returned grey literature search results. Relevant articles were uploaded to Covidence and followed the same screening approach as the database search results. The screening results, along with reasons for exclusion, were reported in the 2020 PRISMA flow diagram [16].

### Data extraction

Following the screening stage, data were extracted from each included study using a pre-determined, data extraction form. The data extraction process was independently pilot tested by three reviewers (CJ, MS and DS) on a sample of included articles (*n* = 5). Data extractors met to discuss discrepancies in the data extraction process and changes were made to the extraction sheet as needed. Data were then extracted from the included articles by one reviewer (CJ) and verified by a second reviewer (MS) to ensure consistency and reliability of results. Weekly team meetings were held during the data extraction phase to discuss any arising concerns with the included articles.

Extracted data included study characteristics such as country, year of publication, study design, objective, population and description of LHS. Any reported details about implementation were also extracted. Qualitative findings were extracted as themes and sub-themes and included concepts related to implementing LHSs, such as stakeholder experiences in how healthcare organizations shifted to a LHS model. Evaluation measures and outcome data were also extracted from qualitative studies when available. Quantitative data related to the implementation and/or evaluation of LHSs were extracted. Quantitative findings included patient-related outcomes, cost effectiveness, provider outcomes, pre-post data, changes in population health and impact on the health system.

### Data synthesis and integration

The LHS details from each study were synthesized using Lavis et al*.* seven LHS characteristics [9]. For example, studies reporting on patient engagement were tagged as such, and an overview of the most and least common LHS characteristics were reported narratively. Similarly, the implementation strategies described by authors were synthesized and categorized using the ERIC taxonomy of implementation strategies [12]. A descriptive synthesis of the most common strategies was reported. Outcome and evaluation details were also synthesized by mapping the reported outcome measures to Allen et al.’s list of 10 outcomes for LHSs [11], while Tierney’s list of implementation outcome measures was used to further categorize how studies reported implementation outcome measures [13]. These details provide a comprehensive picture of how LHSs have been implemented across various health care settings and whether the implementation of LHSs led to changes in health care costs, patient, provider, or population level outcomes.

A convergent integrated approach was used to synthesize the data in this review. This approach involves extracting quantitative and qualitative data separately, followed by an integrated synthesis of all sources of data [14]. First, the findings from quantitative studies were transformed into ‘qualitized’ data. This involved creating a narrative description for each quantitative study's key findings, by extracting the key findings from each study and then reviewing and refining them by two independent reviewers. A similar approach was used with qualitative study findings from included qualitative and mixed methods papers. The final, agreed upon narrative description of key findings from all studies were then integrated using thematic analysis by categorizing and pooling similar findings together. This involved two reviewers independently coding all findings, and then determining a list of common themes. The themes were then refined and finalized through further discussion among three authors, until consensus was reached. A final list of integrated findings was then reported in tables and text.

### Critical appraisal

Included studies were critically appraised by two reviewers (MS and CJ) using the relevant JBI critical appraisal tool, according to study design. The purpose of the tools is to appraise different types of study designs, to provide an objective summary of the design quality. JBI is a reputable organization that specializes in access, appraisal and application of the best available evidence for evidence-based decision making in health and service delivery. The appraisal tool questions can be found in Additional file 1: Table S3. Qualitative studies were appraised using the qualitative checklist and scored out of 10. Cross-sectional studies were scored out of eight, cohort studies were scored out of 11 and quasi-experimental studies were scored out of nine. The reviewers independently appraised each study and then met to discuss final scores. In instances of differently scored items, the reviewers met to discuss their scores until a consensus was reached. A third reviewer was consulted in cases where a decision could not be reached. Final scores were presented as a percentage alongside extracted data in tables, with a detailed overview of study scores presented in a separate table. Grey literature sources and descriptive case studies were not appraised due to a lack of a relevant appraisal tool, and therefore did not receive a critical appraisal score. This was documented in the results tables. In line with mixed methods systematic review methodology [14], no confidence of findings summary table was developed. Due to the heterogeneous nature of mixed methods reviews, it is not recommended to complete this step.

## Results

A total of 5171 studies were identified in the database search, of which 3147 were removed as duplicates. Of the remaining 2024 studies, 27 were deemed relevant and one additional study was found through hand searching reference lists [19]. The grey literature search returned over 700 000 resources, and after reviewing the first five pages of each search, 40 were screened in full text and seven were included in this review. Therefore, a total of 35 resources, describing 31 unique LHSs, were included in this review.

**Fig. 1:**
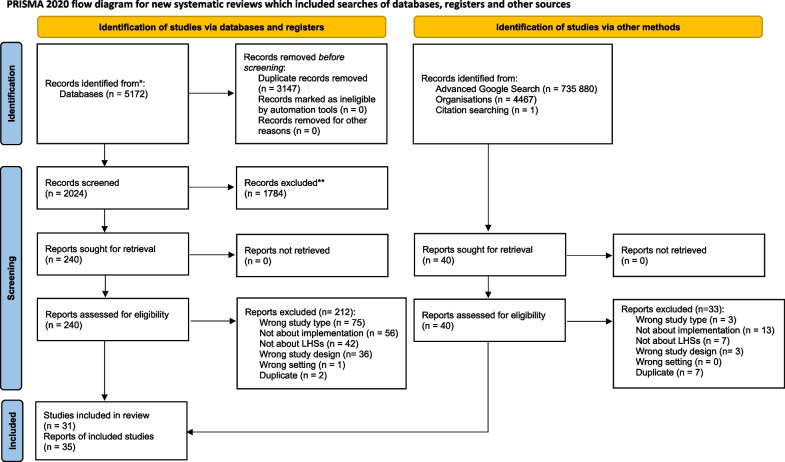
PRISMA flow diagram of included studies. *Consider, if feasible to do so, reporting the number of records identified from each database or register searched (rather than the total number across all databases/registers). **If automation tools were used, indicate how many records were excluded by a human and how many were excluded by automation tools.From:  Page MJ, McKenzie JE, Bossuyt PM, Boutron I, Hoffmann TC, Mulrow CD, et al. The PRISMA 2020 statement: an updated guideline for reporting systematic reviews. BMJ 2021;372:n71. doi: 10.1136/bmj.n71. For more information, visit: http://www.prisma-statement.org/

The main reasons for exclusion included wrong article type (*n* = 78), not related to implementation (*n* = 69), not about LHSs (*n* = 49), wrong study design (*n* = 39), duplicate study (*n* = 9) or not a healthcare setting (*n* = 1). A complete list of the search process can be found in the PRISMA flow diagram (Fig. 1).

### Study characteristics

Of the included studies, the majority were conducted in the United States (US) (*n* = 21) followed by Canada (*n* = 4), the United Kingdom (UK) (*n* = 3), Australia (*n* = 1), Sweden (*n* = 1), Netherlands (*n* = 1), and Europe (*n* = 1). Four studies reported on LHSs implemented across international borders. The date of publication ranged from 2014 to 2022, with most studies (24/35) published between 2019 and 2022. Of the 35 included studies, five were of qualitative design [20–24], four were cross-sectional studies [25–28], two were cohort studies [29, 30], one was quasi-experimental [19] and 23 were descriptive case studies [31–52]. All five of the qualitative studies involved semi-structured interviews and reported on stakeholder views related to implementing LHSs. Together, the qualitative studies described 20 themes related to implementing LHSs. The remaining quantitative and descriptive case studies varied in their designs and outcomes of interest. The case studies described the implementation of LHSs, without reporting on a specific methodological approach to data collection and analysis. The type of health setting varied across studies, with the majority of LHSs implemented in hospitals at a multi-institutional level, often for a specific health condition. The study population varied across studies, with 11 reporting on a pediatric population [26, 28, 29, 35–37, 39, 41, 44, 46, 52], 16 reporting on health system leaders or employees [20–25, 27, 31–34, 43, 45, 49, 50, 53] and eight related to adult populations with various clinical presentations [19, 30, 38, 40, 42, 47, 48, 51]. Study characteristics can be found in Table 1**.**

**Table 1:** Study characteristics and quality score of included papers

Name of LHS	Author, Year (*n* = 35)	Country	Study design	Objective	Health setting	Population/Health condition	CA score
Peds-CHOIR	Bhandari 2016	USA	Cross-sectional Study	To describe the application of CHOIR in a pediatric pain clinic	Pediatric tertiary care pain clinic	Children aged 8–17 years with chronic pain	75%
SPS Network; ICN; NPCQIC; OPQC	Britto, 2018	USA	Case Study	To describe how the LHS organizational framework has been replicated in four conditions, leading to improved outcomes	Multi-institutional network	Various pediatric conditions within 4 LHS	n/a
RCLS-CF	Dixon-Woods 2021	UK	Qualitative	To examine perspectives of health professionals on how to implement a LHS	Multi-institutional network	Providers working with cystic fibrosis patients (*n* = 19)	90%
n/a	Enticott 2020	Australia	Qualitative	To explore features of a LHS	National health organization	Variety of health system stakeholders (*n* = 26)	90%
VA-ESP	Floyd 2019	USA	Cross-sectional Study	To build on previous work, describing LHS needs	Veteran Affairs Health System	Variety of decision-makers (*n* = 66)	25%
PEDSnet & ICN	Forrest 2014	USA	Case Study	To describe a model for a pediatric LHS	Health Network	Inflammatory bowel disease health centres (*n* = 8)	n/a
	Porcaro, 2022	International	Case Study	To describe a strategic governance review of ICN	Health Network	Children and adolescents with IBD across various network sites (*n* = 106)	n/a
The Ottawa Hospital	Fung-Kee-Fung 2018	Canada	Case Study	To describe an approach to improved lung cancer care	Hospital	Patients with lung cancer, providers and caregivers (*n* = 68)	n/a
Neotree	Heys, 2022	International	Case Study	To describe conceptualization, development and implementation experience of Neotree	Multi-institutional network	Newborn care in low-resource settings (*n* = 18 000 babies; *n* = 400 HCPs)	n/a
n/a	Jeffries 2018	UK	Qualitative	To explore how an LHS intervention was implemented in practice	Primary Care	General practice staff & pharmacists (*n* = 22)	70%
SHOnet	Koscielniak, 2022	USA	Case Study	To describe the development of an LHS for a pediatric specialty care rehabilitation network	Multi-institutional Network	Data for over 2 million patient encounters	n/a
MSQC	Krapohl 2020	USA	Case Study	Describe how to implement and sustain a LHS	Multi-institutional Network	Michigan hospitals performing major surgeries (*n* = 70)	n/a
PC-ICCN	Levin, 2022	Canada	Case Study	To describe the PC-ICCN LHS	Multi-institutional Network	Patients living with long COVID (*n* = 5364)	n/a
TRANSFoRm	Lim, 2015	Europe	Case Study	To describe the TRANSFoRm project and the GERD use case	Primary Care	Patients with GERD	n/a
LFEP	Lowes 2017	USA	Cohort Study	To describe the implementation of a LHS for children with cerebral palsy	Hospital	Children with cerebral palsy (*n* = 131)	91%
	Noritz 2018	USA	Cross-sectional Study	To describe the process of screening patients with cerebral palsy for hip displacement	Hospital	Children with cerebral palsy (*n* = 132)	63%
SCK	Miller 2020	USA	Cross-sectional Study	Develop a data dictionary to standardize bedside data entry in real time	Hospital	Patients with sickle cell disease (*n* = 285)	75%
IDEA4PS	Moffat-Bruce 2018	USA	Case Study	Describe the experience of an academic medical center in developing an LHS	Hospital	Stakeholders from more than 8 investigators (*n* = 23)	n/a
MS PATHS	Mowry 2020	International	Cohort Study	To describe initial implementation of MS PATHS	Multi-institutional Network	Patients with multiple sclerosis from 10 institutions (*n* = 16 568)	55%
ATN/AIR-P	Murray 2019	USA	Case Study	To describe the transition to a learning network, including lessons learned	Multi-institutional Network	Network sites providing autism care (*n* = 12)	n/a
The Alliance for Healthier Communities	Nash, 2022^a^	Canada	Qualitative	Provide guidance for health systems to develop a LHS	Primary Care	Stakeholders (*n* = 29) from 6 community health centres	60%
	Nash, 2022^b^	Canada	Case Study	To describe the process of developing a province-wide LHS in primary care	Multi-institutional Network (Primary Care)	Community-governed primary care organizations (*n* = 109)	n/a
SNEPT	Perito 2021	USA	Case Study	To describe the creation of a multicenter LHS for pediatric liver transplantation	Multi-institutional Network	Health care sites providing pediatric liver transplant care (*n* = 10)	n/a
myAva	Satveit 2018	USA	Case Study	To describe the myAva program as a LHS for women with polycystic ovarian syndrome	Health Network	Patients living with polycystic ovarian syndrome (PCOS) (*n* = 55)	n/a
ClaudicatioNet Quality System	Sinnige, 2021	Netherlands	Case Study	To describe the transformation of ClaudicatioNet into an LHS which aims to improve physical therapy care for patients with intermittent claudication	Multi-institutional Network	Patients with intermittent claudication	n/a
CHC	Steels 2021	UK	Qualitative	To present preliminary findings of implementing the CHC LHS across the north of England	Multi-institutional Network	CHC program staff and external partners (*n* = 59)	60%
CORE	Taylor 2021	USA	Case Study	To describe the health system transformation to a LHS	Multi-institutional Network	Various health system concerns	n/a
EQUIPPED	Vandenberg 2020	USA	Case Study	To describe the implementation of the EQUIPPED medication safety program	Multi-institutional Network	Team members from three implementation emergency department sites (*n* = 18)	n/a
	Vaughan 2021	USA	Quasi-experimental Study	To describe prescribing behaviours following implementation of the EQUIPPED medication safety program	Multi-institutional Network	Adults aged 65 years and over being discharged from emergency department sites	56%
n/a	Varnell, 2022	USA	Case Study	To describe how building improved health systems with integrated clinical care as part of an LHS can improve pediatric nephrology care	Hospital	Kidney transplant patients from one clinical department	n/a
Grey Literature Sources							
Baylor Scott and White Health	AHRQ, 2019	USA	Case Study	To describe the transition of the Baylor Scott & White Health system to an LHS	Multi-institutional network	Multiple healthcare institutions (*n* = 967)	n/a
Denver Health	AHRQ, 2019	USA	Case Study	To describe the transition of the Denver Health system to an LHS	Multi-institutional network	Multiple healthcare institutions (*n* = 29)	n/a
HCA Healthcare	AHRQ, 2019	International	Case Study	To describe the transition of HCA Healthcare to an LHS	Multi-institutional network	Multiple healthcare institutions (*n* = 304)	n/a
University of Utah Health	AHRQ, 2019	USA	Case Study	To describe the transition of the University of Utah Health system to an LHS	Multi-institutional network	Multiple healthcare institutions (*n* = 42)	n/a
Geisinger Health System	Foley, 2015	USA	Case Study	To describe GHS and its approach to becoming an LHS	Multi-institutional network	Integrated health services organization serving general public (*n* = 2.6 million patients)	n/a

*AHRQ* Agency of Healthcare Research and Quality, *ATN/AIR-P* Autism Treatment Network & Autism Intervention and Research Network on Physical Health, *CA* Critical Appraisal, *CHC* Connected Health Cities, *CHOIR* Collaborative Health Outcomes Information Registry, *CORE* Center for Outcomes Research and Evaluation, *COVID* Coronavirus Disease, *EQUIPPED* Enhancing Quality of Prescribing Practices for Older Adults in the Emergency Department, *GERD* Gastroesophageal reflux disease, *GHS* Geisinger Health System, *HCA* Hospital Corporation of America, *IBD* Inflammatory bowel disease, *ICN* Improve Care Now, *IDEA4PS* Institute for the Design of Environments Aligned for Patient Safety, *LFEP* learn from every patient, *LHS* Learning Health System, *MS PATHS* Multiple Sclerosis Partners Advancing Technology and Health Solutions, *MSQC* Michigan Surgical Quality Collaborative, *NPCQIC* National Pediatric Cardiology Quality Improvement Collaborative, *PCICCN* Post COVID-19 Interdisciplinary Clinical Care Network, *OPQC* Ohio Perinatal Quality Collaborative, *Peds-CHOIR* Pediatric adaptation of the Collaborative Health Outcomes Information Registry, *RCLS-CF* Registry-enabled Care and Learning System for Cystic Fibrosis, *SCK* Sickle cell knowledge base, *SHOnet* Shriners Hospitals for Children (SHC) Health Outcomes Network, *SPS* Solutions for Patient Safety, *SNEPT* Starzl Network for Excellence in Pediatric Transplantation, *UK* United Kingdom, *USA* United States of America, *VA-ESP* Veterans Affairs Evidence Synthesis Program

### Learning health system characteristics

Synthesis of LHS characteristics revealed that of the 31 unique LHSs (reported across 35 studies) eight included all seven of Lavis et al*.* characteristics [35, 36, 41, 42, 44, 45, 47, 53]. Two studies [25, 52] only reported two LHS characteristics, and these included the ‘digital data capture’, ‘timely production of evidence’ and ‘appropriate decision supports’ characteristics. Digital data capture was reported most often, in 31 studies. Examples of this characteristic included when a LHS incorporated an electronic health record (EHR) as part of the system or the use of dashboards and databases to enable data sharing. Patient engagement, aligned governance, and the culture of rapid learning and improvement characteristics were reported least often, in 21 studies each. Table 2 outlines the number and types of LHS characteristics reported across studies. A more detailed description of the LHS constructs identified in each study can be found in Additional file 1: Table S4.

**Table 2:** Learning health system characteristics based on Lavis et al.’s seven characteristics

LHS Name (reference/s)	Author, year (*n* = 35)	LHS constructs							Number of reported constructs (*n*/7)
		Patient engagement	Digital capture	Timely production of evidence	Appropriate decision supports	Aligned governance	Culture of rapid learning & improvement	Culture of competencies enabled	
Peds-CHOIR	Bhandari, 2016	Yes	Yes	Yes	No	No	Yes	Yes	5
SPS Network; ICN; NPCQIC; OPQC	Britto, 2018	Yes	Yes	Yes	Yes	Yes	Yes	Yes	7
RCLS-CF	Dixon-Woods, 2020	Yes	Yes	Yes	Yes	No	Yes	Yes	6
n/a	Enticott, 2020	Yes	Yes	Yes	Yes	Yes	No	Yes	6
VA-ESP	Floyd, 2019	No	No	Yes	Yes	No	No	No	2
PEDSnet & ICN	Forrest, 2014	Yes	Yes	Yes	Yes	Yes	Yes	Yes	7
	Porcaro, 2022	Yes	Yes	No	No	Yes	No	No	3
The Ottawa Hospital	Fung-Kee-Fung, 2018	Yes	Yes	Yes	Yes	No	No	Yes	5
Neotree	Heys, 2022	No	Yes	Yes	Yes	Yes	Yes	Yes	6
n/a	Jeffries, 2018	No	Yes	Yes	Yes	No	No	Yes	4
SHOnet	Koscielniak, 2022	Yes	Yes	Yes	Yes	Yes	Yes	Yes	7
MSQC	Krapohl, 2020	Yes	Yes	Yes	Yes	Yes	Yes	Yes	7
PC-ICCN	Levin, 2022	Yes	Yes	Yes	Yes	Yes	Yes	Yes	7
TRANSFoRm	Lim, 2015	Yes	Yes	Yes	No	No	No	No	3
LFEP	Lowes, 2017	No	Yes	Yes	Yes	No	No	No	3
	Noritz, 2018	Yes	Yes	Yes	Yes	No	No	No	4
SCK	Miller, 2020	No	Yes	Yes	Yes	No	No	Yes	4
IDEA4PS	Moffat-Bruce, 2018	No	No	No	No	Yes	Yes	Yes	3
MS PATHS	Mowry, 2020	Yes	Yes	No	Yes	Yes	Yes	Yes	6
ATN/AIR-P	Murray, 2019	Yes	Yes	Yes	Yes	Yes	Yes	Yes	7
Alliance for Healthier Communities	Nash, 2022^a^	N/a	N/a	N/a	N/a	N/a	N/a	N/a	n/a
	Nash, 2022^b^	Yes	Yes	Yes	Yes	Yes	Yes	Yes	7
SNEPT	Perito, 2021	Yes	Yes	Yes	No	Yes	Yes	Yes	6
myAva	Satveit, 2018	Yes	Yes	Yes	Yes	Yes	Yes	Yes	7
ClaudicatioNet Quality System	Sinnige, 2022	Yes	Yes	No	Yes	No	Yes	Yes	5
CHC	Steels, 2021	Yes	Yes	No	No	Yes	Yes	Yes	5
CORE	Taylor, 2021	No	Yes	No	No	Yes	Yes	Yes	4
EQUIPPED	Vandenburg, 2020	No	Yes	No	Yes	Yes	No	Yes	4
	Vaughan, 2021	n/a	n/a	n/a	n/a	n/a	n/a	n/a	n/a
n/a	Varnell, 2022	No	Yes	No	Yes	No	No	No	2
Grey Literature (*n* = 5)									
Baylor Scott and White Health	AHRQ, 2019	No	Yes	Yes	Yes	Yes	Yes	Yes	6
Denver Health	AHRQ, 2019	No	Yes	Yes	Yes	Yes	Yes	Yes	6
HCA healthcare	AHRQ, 2019	No	Yes	Yes	Yes	Yes	Yes	Yes	6
University of Utah Health	AHRQ, 2019	Yes	Yes	No	Yes	Yes	Yes	Yes	6
Gesinger Health System	Foley, 2015	Yes	Yes	No	Yes	No	No	No	3
Totals		21/35	31/35	23/35	26/35	21/35	21/35	26/35	

### Implementation strategies

All study authors reported on the implementation of a LHS, including the specific implementation strategies used, and/or implementation outcomes. Of ERIC’s taxonomy of 73 strategies, 33 different implementation strategies were used across studies. The most common implementation strategies were change record systems (*n* = 20) [22, 26, 28–36, 38, 40, 45, 46, 48, 50–53], conduct local consensus discussions (*n* = 7) [19, 28, 29, 32, 37, 39, 49], audit and provide feedback (*n* = 6) [19, 22, 25, 43, 50, 53], build a coalition (*n* = 5) [20, 26, 41, 44, 46], and develop and organize quality monitoring systems (*n* = 5) [21, 27, 35, 44, 46]. Fourteen strategies were reported only once across studies. Table 3 provides an overview of the reported implementation strategies with an example from select papers.

**Table 3:** Overview of identified implementation strategies based on the ERIC taxonomy

Implementation strategy (*n* = 33)	Number of studies	Select example from papers
Change record systems	20 (22,26,28–36,38,40,45,46,48,50–53)	Expand the CHOIR system for tracking of patient/caregiver data (Bhandari, 2016) Automated workflows in EHRs to improve patient flow (Fung-kee-fung, 2018)
Conduct local consensus discussions	7 (19,28,29,32,37,39,49)	Create early wins and get buy-in from health system leaders (Taylor, 2021) Local stakeholders were employed which helped with community engagement and provided essential local expertise and leadership (Heys, 2022)
Audit and provide feedback	6 (19,22,25,43,50,53)	Dashboard with safety indicators allows pharmacists to identify high risk patients and potential hazardous prescribing (Jeffries, 2018) Translates performance to data, data to knowledge and knowledge to performance to reduce opioid prescribing for surgical patients (Kraphol, 2020)
Build a coalition	5 (20,26,41,44,46)	Build capacity for clinicians to provide evidence-based ASD care (Murray, 2019) Aim to establish partnerships with clinicians, researchers, patients, families and other health system stakeholders (Koscielniak, 2022)
Develop and organize quality monitoring systems	5 (21,27,35,44,46)	Quality checks are continuously implemented when a patient visits and is discharged from hospital (Miller, 2020) A cyclic improvement process was noted as being important to ensure clinicians were entering high quality data (Enticott, 2020)
Use advisory boards and workgroups	4 (33,39,42,48)	ClaudicatioNet is run by multiple stakeholders with various backgrounds and a range of knowledge, expertise and education (Sinnige, 2022) Implementation was successful due in-part to the strong African clinical leadership and buy-in from the Ministry of Health (Heys, 2022)
Create a learning collaborative	4 (38,43,45,49)	Clinicians, researchers, analytics staff, health services experts, and information technology teams work together to improve operational excellence (Moffat-Bruce, 2018)
Promote network weaving	4 (31,33–35)	At HCA Healthcare, projects are pilot tested at one facility and then expanded across facilities if successful. This helps to ensure success and sustainability of the implemented projects (AHRQ, 2019)
Remind clinicians	4 (31,32,48,50)	The EHR at Denver Health was designed to include reminders for clinicians for certain screening tests with the daily appointment schedule (AHRQ, 2019) Data is leveraged in several ways at Geisinger Health, including incorporating dashboards for prompts and including automation where possible (Foley, 2015)
Change physical structure and equipment	3 (19,31,35)	Leaders at Baylor Scott and White Health identified a need to implement structures to support innovation in order to use data to make decisions (AHRQ, 2019)
Conduct cyclical small tests of change	3 (21,46,49)	Pragmatic, mixed methods study designs needed to facilitate research to inform system change in a LHS (Taylor, 2021)
Facilitate relay of clinical data to providers	3 (25,44,47)	The data reports were most often requested for informing clinical guidance, determining future research priorities and identifying relevant implementation strategies (Floyd, 2019)
Promote adaptability	3 (19,32,51)	Implementation of EQUIPPED requires site-specific adaptability (Vandenberg, 2020)
Conduct ongoing training	2 (26,35)	Training implemented to help health centres identify and onboard patients to the improvement team (Britto, 2018)
Develop academic partnerships	2 (42,49)	Research scholars have been hired to produce outputs to inform care and identify research questions (Levin, 2022)
Develop educational materials	2 (42,48)	ClaudicatioNet communicates frequently about the progression of projects to create early awareness and has developed instructional videos to enhance the accessibility of information (Sinnige, 2022)
Identify and prepare champions	2 (19,45)	Clinicians and front-line staff are driving change within the practice-based learning network from the bottom-up (Nash, Brown, et al. 2022)
Involve patients & consumers to enhance uptake & adherence	2 (26,42)	Conversations with patients to understand highlights and optimize patient care (Bhandari, 2016) Experienced patient research partners and individuals who were new to patient engagement were involved from the beginning to ensure a patient oriented approach was used (Levin, 2022)
Use data experts	2 (30,33)	Each institution directly negotiated with Biogen to simplify the data sharing process (Mowry, 2020)
Use data warehousing techniques	2 (24,41)	A data warehouse developed based on standard terminology, data model and elements (Kosclieniak, 2022)
Access new funding	1 (26)	Cost of implementing Peds-CHOIR into the clinic and annual maintenance (Bhandari, 2016)
Conduct educational meetings	1 (26)	Implementation of staff training and education for clinicians around how the LHS can be used for patient care (Bhandari, 2016)
Conduct educational outreach visits	1 (19)	Provider feedback process involved 1–1 sessions with a local EQUIPPED champion, site visits and reports given to providers within 3 months of implementing the program (Vaughan, 2022)
Create new clinical teams	1 (34)	There was an investment in building teams to analyze data and deliver information for use by various parts of the health system (AHRQ, 2019)
Develop and implement tools for quality monitoring	1 (20)	Develop the dashboard that reflects patients’ goals, treatment and outcomes (Dixon-Woods, 2020)
Facilitation	1 (42)	An education program and provincial help-line were established to support physicians in implementing the LHS (Levin, 2022)
Purposely re-examine the implementation	1 (21)	It was identified that a cyclic improvement process where clinicians were made aware of the importance of data outcomes for patient care was key to ensuring quality data entry (Enticott, 2020)
Obtain and use patients/consumers and family feedback	1 (36)	PCORnet will include data from millions of Americans which will help to learn what works for which patients (Forrest, 2014)
Change service sites	1 (38)	A centralized regional process established for review and processing of lung cancer referrals (Fung-kee-fung, 2018)
Mandate change	1 (49)	The vision for CORE began with research leaders who recognized the potential of Atrium Health on improving patient outcomes (Taylor, 2021)
Provide clinical supervision	1 (38)	System involved creation of a standardized process which included a physician order sheet and clinical patient note to assist in daily case reviews (Fung-kee-fung, 2018)
Provide ongoing consultation	1 (38)	The LHS involved interdisciplinary consults with lung cancer specialists for all referrals received (Fung-kee-fung, 2018)
Recruit, designate and train for leadership	1 (44)	Patient-family navigators were recruited and trained to provide support to families so they could access resources (Murray, 2019)

### Study outcomes

Systematic adoption of evidence-based practices (EBP) was the LHS outcome reported most often (*n* = 17) according to Allen et al.’s list, followed by knowledge to action latency (*n* = 12) and population health (*n* = 12). Using Tierney’s list of implementation measures, the majority of studies commented on intervention complexity (*n* = 16) and adoption (*n* = 13). Only two studies spoke about implementation cost [28, 29] and no studies discussed fidelity as part of their LHS implementation approach. Based on the quadruple aim (patient, provider, population and health care cost), 23 studies reported on outcomes related to patients [19–21, 24, 26, 28–36, 38, 39, 44, 46–48, 50, 52, 53], eight studies addressed provider outcomes [20, 22–25, 27, 39, 43], 15 studies were related to population-level outcomes [26, 27, 36–42, 45, 46, 48, 49, 51, 53] and five studies reported on healthcare costs [28, 29, 31, 34, 35]. Table 4 provides an overview of study outcomes.

**Table 4:** Overview of outcome data including patient, provider, population and health system cost outcomes and implementation outcomes

LHS name	Author, Year	Allen’s list of LHS outcomes (11)	Implementation measures (13)	Impact outcomes	Outcome data	Key finding(s)
Quantitative studies						
Peds-CHOIR	Bhandari 2016	Population health	Adoption Penetration	Patient Population	Peds-CHOIR allowed for faster decision-making and trialing of interventions based on patient and caregiver feedback	Peds-CHOIR is an example of a platform that highlights predictors of chronic pain and enables individually tailored interventions/treatment
VA-ESP	Floyd, 2019	Knowledge to action latency; Systematic adoption of EBP	Acceptability Adoption Appropriateness Feasibility	Provider	Evidence synthesis reports most often requested to inform clinical guidance (58%), identify future research needs (58%), and determine implementation strategies (47%) 91% of end-users used the evidence reports within 3 months of completion (82%) Evidence reports most often used to inform policy or guidance (26%) and inform procurement decisions (21%)	VA-ESP evidence products can inform clinical practice and policy and are often used within 3 months of completion by decision makers
LFEP	Lowes 2017	Knowledge to action latency; Systematic adoption of EBP; Care experience; Programmatic return on investment	Feasibility Implementation cost	Patient Cost	43% reduction in inpatient days 27% reduction in inpatient admissions 29% reduction in urgent care visits 176%-210% reduction in healthcare costs	LHS can be implemented to rapidly integrate evidence into clinical practice in a cost-effective way
	Noritz, 2018	Systematic adoption of EMP; Systematic elimination of wasteful and ineffective practices	Implementation cost	Patient Cost	Patient radiation exposure was reduced Annual costs were reduced to an average of $66 per x-ray per child	Implementation of a local LHS allowed for integration of evidence into practice leading to improved patient care and reduced costs
SCD	Miller, 2020	Knowledge to action latency Systematic adoption of EBP	Adoption Feasibility	Provider Population	SCD providers entered dates for clinical visits correctly 99% of the time The LHS allowed for the collection of population health data to inform clinical knowledge	The SCD LHS allowed for data collection at the bedside and timely integration of data into clinical records
MS PATHS	Mowry, 2020	Systematic adoption of EBP; Population health	Feasibility Intervention complexity	Patient	The MS PATHS LHS enrolled over 16 500 participants, with 88.4% providing data for at least one time point and the average contribution of 15.6 person-months	MS PATHS is an example of how an MS practice can collect and integrate patient data to inform clinical decisions and continuous learning
Descriptive case studies						
Baylor Scott & White Health	AHRQ, 2019	Knowledge to action latency; Systematic adoption of EBP; Programmatic return on investment	Penetration Reach	Patient Cost	Over 5 years, the model has led to $280 million in savings while improving patient outcomes	Baylor Scott & White Health have prioritized learning & knowledge generation by focusing on data infrastructure, organizational culture and supporting a continuous cycle of improvement
Denver Health	AHRQ, 2019	Knowledge to action latency; Systematic adoption of EBP	Adoption Penetration Reach	Patient	Reduced surgical infection rates due to Denver Health’s culture of learning; Improved cancer screening rates due to digital infrastructure interventions	Denver health provides a model for how a LHS can provide higher quality, safer and more efficient care
HCA	AHRQ, 2019	Knowledge to action latency; Systematic adoption of EBP	Adoption Penetration	Patient	Improved time to biopsy results for patients; Improved SPOT system led to quicker identification and survival rates of sepsis patients;	HCA's LHS exemplifies how a large for-profit health system can use its resources to support health system transformation using a strong foundation of data and continuous learning
University of Utah Health	AHRQ, 2019	Care experience; Programmatic return on investment	Intervention complexity Penetration	Patient Cost	Health system leaders created a Resident Value Council to support resident training in quality, safety, efficiency and workflow The University of Utah Health Care Partners program is an initiative that led to the approval of 65 projects and a cost savings of $8.6 million	University of Utah Health provides a strong model for how an LHS can function by investing in value-based care, having sophisticated data operation and a culture and workforce dedicated to continuous improvement
Learning Networks	Britto, 2018	Systematic adoption of EBP; Population health	Intervention complexity Penetration	Patient Cost	The ICN LHS resulted in an 80% clinical remission rate The NPCQIC LHS led to 40% reduced mortality among patients The SPS resulted in a reduction of several hospital acquired conditions by 5%-79% The OPQC LHS led to improved outcomes across multiple areas of patient care Several LHSs invest in the program due to the observed financial benefits to the system and patients	The LHS networks described in this paper are examples of replicable LHSs and have led to improved patient outcomes across multiple diseases and patient populations
Geisinger Health System	Foley, 2015	Systematic elimination of wasteful & ineffective practices	Intervention complexity	Patient	Reduction in no-shows from 47 to 24% as a result of using predictive data modeling	GHS developed innovative analytic techniques to capture data and recognizes that patient engagement with information is key in improving patient experiences and outcomes. Challenges identified in fully realizing the LHS
PEDSnet & ICN	Forrest, 2014	Systematic adoption of EBP Population health	Adoption Penetration Reach	Patient Population	Since its inception in 2007, ICN has grown from 8 to 66 GI care centres across the USA ICN increased remission rates from 55 to 77%	Based on the success of ICN for pediatric patients with inflammatory bowel disease, a national LHS, PEDSnet, will be scaled up and spread across health care organizations and patient populations
	Porcaro, 2022	Programmatic return on investment	Intervention complexity	Population	n/a	LHS networks can grow with supportive governance that is flexible to changing technology and stakeholder needs
The Ottawa Hospital	Fung-Kee-Fung, 2018	Systematic adoption of EBP Population health	Adoption Feasibility Penetration Reach Sustainability	Patient Population	Time to diagnosis within 14 days was improved & above provincial target timeline Diagnosis made to 80% of referrals within 28 days, versus 57% for the province Time from referral to 1st treatment decreased from 92 to 47 days (by 48%) Diagnosed patients receiving no treatment decreased from 22 to 16% over 2 years	The implementation of an LHS for cancer patients in Ottawa led to improved time from referral to initial treatment and was sustained over time
Neotree	Heys 2022	Systematic adoption of EBP; Population health; Equity	Acceptability Adoption Appropriateness Feasibility Intervention complexity Penetration Reach	Patient Provider Population	Reduction from 79 to 38% admission rate of hypothermic babies Unnecessary antibiotic prescribing fell from 97 to 2% Improved health care provider confidence & ability to provide newborn care	Initial development of a digital QI system for newborn care shows potential for a sustainable LHS in low resource settings. Neotree is an ongoing project with additional implementation, evaluation and outcome data to be published in future
SHOnet	Koscielniak, 2022	Knowledge to action latency	Feasibility	Population	The SHOnet LHS includes data from over 2 million patient encounters over 10 years	SHOnet provides an example of an LHS for pediatric rehabilitation settings, where data are extracted from EHRs and integrated into clinical care
MSQC	Krapohl, 2020	Knowledge to action latency; Systematic adoption of EBP; Care experience	Adoption Sustainability	Patient Population	Reduced opioid prescribing Less variability between providers across the collaborative	LHS principles can accelerate the translation of evidence into practice and improve patient outcomes
PC-ICCN	Levin, 2022	Systematic elimination of wasteful and ineffective practices; Population health	Appropriateness Penetration	Population	To date, 5364 patients have been referred and 2354 patients have visited the clinic at least once	PC-ICCN is an LHS that allows for the integration of data into clinical care across a provincial program
TRANSFoRm	Lim, 2015	Population health	Intervention complexity	Population	n/a	TRANSFoRm is an example of an LHS that integrates research into clinical practice by working with the EHR as a data collection system
IDEA4PS	Moffat-Bruce, 2018	Systematic adoption of EBP	Acceptability Intervention complexity	Provider	Three pilot studies implemented as part of IDEA4PS led to improvements: Implementation of a falls wheel was effective in engaging patients, clinicians and researchers & improved falls safety; Patient safety indicators study demonstrated how research could improve processes & practices; A telemetry and alarms study led to reduced telemetry days and increased ED throughput	By reframing the role of research in improving outcomes, IDEA4PS has allowed for capacity building and the development of a learning culture
ATN/AIR-P	Murray, 2019	Care experience	Acceptability Adoption Sustainability	Patient	As of 2018, 731 patients were enrolled in the network Parents identified priorities for clinical care	The ATN/AIR-P is an example of how infrastructure enabled improvement and research and allowed for systematic collection of clinical data to inform practice
Alliance for Healthier Communities	Nash, 2022^b^	Systematic adoption of EBP	Acceptability	Population	Preliminary outcomes indicate improved cancer screening and equity across marginalized groups, for a sub-group of the LHS, indicating future potential impact of the broader AHC Over 70 stakeholders actively involved in co-creating the LHS	The LHS success can be attributed to a positive organizational culture, supportive leadership, an EHR that allows for digital data capture, motivated providers and staff, and resources for data support
SNEPT	Perito, 2021	Knowledge to action latency; Population health	Appropriateness Feasibility Sustainability	Patient Population	In 2 years, SNEPT built a network that integrates family and stakeholder input, supports transparency and data-sharing efforts, and includes multicenter collaboration for improved pediatric liver transplantation	Pediatric liver transplantation care can be advanced through implementing a LHS and leveraging patient engagement, big data, technology and thought leaders to address most challenging issues
myAva	Satveit, 2017	Systematic adoption of EBP	Acceptability Adoption	Patient	The myAva platform highlighted a need for better PCOS care; MyAva allowed for more patient empowerment and engagement, but revealed challenges in payment structure and provider knowledge	Significant improvements noted after implementing the myAva platform with expectations for continued improvement in PCOS care overtime
ClaudicatioNet	Sinnige, 2022	Knowledge to action latency; Population health; Care experience	Intervention complexity Penetration	Patient Population	ClaudicoNet is a national network of over 2100 physical therapists and includes a registry of routinely collected patient data, which is used to inform care	The Claudicatio Net LHS is an example of how physical therapists can facilitate continuous learning and integrate clinical data into patient care
CORE	Taylor, 2021	Knowledge to action latency	Intervention complexity	Population	CHOSEN antibiotic stewardship case study led to 10% decrease in inappropriate prescribing & a new process for tracking inappropriate prescribing	Case studies exhibit how practice informs research and research informs practice change in an LHS model
EQUIPPED	Vandenberg, 2020	Systematic adoption of EBP	Adoption Feasibility Sustainability	Population	Significant reduction in prescribing potentially inappropriate medications at one site and benzodiazepine prescriptions reduced at all sites, 12-months after implementation	EQUIPPED is feasible to implement and shows promise in reducing inappropriate medication prescribing
	Vaughan 2021	Knowledge to action latency; Population health	Adoption Intervention complexity Sustainability	Patient	Following implementation of EQUIPPED, one site showed a statistically significant decrease in PIMs prescribing rates, while two sites showed no difference	EQUIPPED is a model for addressing medication safety through sequential implementation, with opportunities for scale and spread in other community-based settings
n/a	Varnell, 2022	Population health	Intervention complexity	Patient	Cholesterol level checks increased from 84 to 95% Number of dyslipidemia patients on statins increased from 52 to 88% Number of patients with healthy LDL level went from 65 to 83% Improved rates of rejection-free transplants from 80 to 90% Additional improvements in biochemical markers were observed	This is an example of how a learning health network can be implemented and improve outcomes for a pediatric nephrology population
Qualitative studies						
RCLS-CF	Dixon-Woods, 2020	Work life for care teams	Appropriateness Intervention complexity	Patient Provider	Although all stakeholders shared the same vision and values for the LHS, there are challenges in implementation related to the social and technical aspects of LHSs	
	Themes:	T1: Design stakeholders’ views of the foundations of RCLS-CF (co-production and its transformational potential)—every clinical interaction and patient input could generate meaningful knowledge to improve patient care and decision making T2: Design stakeholders’ views of the technical prerequisites for the learning system—to facilitate more co-produced care plans, need a dashboard that could be viewed by both patients and clinicians T3: Design stakeholders’ views of the social conditions necessary for program implementation—social, cultural and practical barriers identified in gaining universal LHS buy-in from clinicians and patients T4: Design stakeholders’ views of tensions and challenges in implementing the LHS—possible tensions identified in inhibiting the implementation of the LHS, however they can be managed				
n/a	Enticott, 2020	Work life for care teams	Intervention complexity Sustainability	Patient	Structure, governance, trust, culture, vision, leadership and quality data access seen as crucial to implementing and sustaining a LHS	
	Themes:	T1: Systematic approaches and iterative, continuous learning with implementation into healthcare contributing to new best-practice care T2: Broad stakeholder, clinician and academic engagement, with collective vision, leadership, governance and a culture of trust, transparency and co-design T3: Skilled workforce, capability and capacity building T4: Resources with sustained investment over time T5: Data access, systems and processes being integral to a sustainable LHS				
n/a	Jeffries, 2018	Work life for care teams	Acceptability Feasibility	Provider	There is value in integrating information technology and pharmacists in the general practice setting to optimize safe medication administration	
	Themes:	T1: Coherence – dashboard was perceived as easy, valuable and able to integrate by working with other staff T2: Cognitive participation – mixed engagement from different stakeholders but by leading the work, pharmacists were able to show value of the intervention T3: Collective action – communication & collaboration between practitioners was key to success T4: Reflexive monitoring – pharmacists helped improve features of the dashboard, which was a tool seen to enhance patient care & changes in work				
Alliance for Healthier Communities	Nash, 2022 (a)	Work life for care teams	Appropriateness Intervention complexity	Provider	Key elements needed to establish a LHS in primary care include having a positive organizational culture and supportive leadership, an integrated data entry system, motivated providers and staff with capacity to engage with the LHS and access to resources to support LHS initiatives and data collection	
	Themes:	T1: Shared organizational goals and culture—viewed as important for a functioning LHS and this was already in place in some community health centres T2: Data quality—good data quality was identified as necessary for a LHS T3: Resources—limited time for data entry and quality improvement was a barrier T4: People—having leadership who supports LHS is important, but some centres face resistance from staff T5: Motivation—different reasons for motivation among staff to adopt a LHS model, such as improving patient care and making work load more efficient				
n/a	Steels, 2021	Work life for care teams	Intervention complexity	Patient Provider	This paper outlines the challenges in implementing a LHS in Northern England, while also highlighting that this work led to building IT and health informatics infrastructure across NHS organizations	
	Themes:	T1: Challenges in the implementation of LHS pathways—Several challenges identified as sub-themes including time constraints, data access, long-term sustainability and commitment, different working cultures and priorities and communication T2: Benefits to the CHC approach for both staff and patients—Several benefits identified as sub-themes including benefits for staff involved in the CHC program and patients of the CHC program activities				

### Integrated findings

The key findings reported across studies were heterogeneous in nature and therefore a meta-analysis was not possible. Rather, the main findings from all studies were integrated and thematically organized. Quantitative outcome data was qualitized and pooled along with the qualitative and descriptive study outcomes to reveal three main integrated findings and six sub-findings. These integrated findings are described in text below and in Table 5.

**Table 5:** Overview of integrated study findings along with sub-findings and supporting examples from the literature

Integrated finding with description	Sub-findings	Number of studies	Example findings
*The digital infrastructure of LHSs optimizes health service delivery*: Studies reported the implementation of LHSs as having an impact on clinical practice as a result of incorporating a digital infrastructure for data capture as part of their LHS	LHSs can allow for better integration of data/evidence into clinical practice	15 (25,26,30,31,39–42,44,46,48–50,52,53)	Evidence and research from the LHS was integrated into clinical care (25) MS PATHS is an example of how an MS practice can collect and integrate patient data to inform clinical decisions (30)
	LHSs promote the implementation of digital data capture	16 (19,21–24,31,32,39–41,44–47,50,51)	TRANSFoRm is an example of a LHS that integrates research into practice by working with the EHR as a data collection system (40)
	Access and availability of data through a digital platform supports rapid changes to practice and policy	7 (25–29,33,38)	Decision makers used evidence synthesis reports within a short time frame to inform decisions, practice and policy (25) The LHS led to improved time for biopsy results for patients (33)
*LHSs have a positive impact on patient care and health outcomes*: Several studies reported seeing positive patient outcomes following the implementation of a LHS	n/a	17 (19,22,28,29,32,33,35–39,42,43,45,49,51,52)	LFEP resulted in reduced inpatient days, overall admissions and urgent care visits (28,29) Patients with lung cancer received a quicker diagnosis, referral and treatment. Percentage of patients receiving treatment improved overall (38)
*LHSs can influence health care providers and the health system:* The implementation of LHSs appear to impact the culture and experience of health care providers and staff, while also leading to cost savings for some health systems	Implementation of a LHS may help foster a culture of learning and improvement for sustained success	8 (21,23,31,33,34,39,43,45)	Positive organizational culture, supportive leadership and integrated data entry system are crucial for establishing a successful LHS in primary care (23,45) Reframing the role of research in improving outcomes allowed for development of a learning culture (43)
	Health system leaders identify challenges in implementing LHSs, despite recognizing their value	4 (20,24,47,50)	All stakeholders shared the same vision for the LHS, yet they identified challenges in its implementation (20) Stakeholders identified several challenges in implementing a LHS across organizations in Northern England (24)
	LHSs may result in cost savings for the health system	5 (28,29,31,34,35)	Baylor Scott & White Health led to $280 million in savings (31)

#### Integrated finding 1: the digital infrastructure of LHSs optimizes health service delivery

Multiple studies reported on how the implementation of a LHS impacted health service delivery, such as by allowing for the rapid inclusion of evidence into practice or by providing an infrastructure to support digital data capture. Three categories were grouped under this main finding. The first describes how LHSs can allow for better integration of data and evidence into clinical practice. Fifteen studies aligned with sub-finding 1a and reported on how the implementation of a LHS can allow for better integration of data and evidence into practice, such as having a platform that highlights chronic pain in patients to inform care [26] or a database with information about patients with multiple sclerosis that clinicians use to inform decisions [30]. Sub-finding 1b includes how LHSs promote the implementation of digital data capture. Sixteen studies described how a LHS can provide an opportunity for digital data capture [19, 21–24, 31, 32, 39–41, 44–47, 50, 51]. In some cases, the digital infrastructure was not available or valued prior to the implementation of a LHS, but with the health system transformation, systems were able to see how infrastructure could support the collection of digital data. This was often viewed as key to the LHS implementation process. Sub-finding 3c describes that access and availability of data through a digital platform supports rapid changes to practice and policy. Seven studies aligned with this finding and described how the LHS allowed for faster changes to practice and policy [25–29, 33, 38]. In some studies, this was a result of having electronic data more readily available to practitioners, allowing for rapid decisions and changes to patient care.

#### Integrated finding 2: learning health systems have a positive impact on patient care and health outcomes

The second integrated finding is represented by 17 studies [19, 22, 28, 29, 32, 33, 35–39, 42, 43, 45, 49, 51, 52] which reported positive patient outcomes as a result of implementing a LHS. These studies included varied patient outcomes such as improved diagnosis, screening or referral rates [38], reduced readmission rates and decreased length of stay [29], and improved prescribing practices [19, 39, 51, 53].

#### Integrated finding 3: learning health systems can influence health care providers and the health system

The third main finding is related to the impact of LHSs on the health system, including the physical environment and people within the system. This finding included three sub-findings. The first sub-finding, 3a, states how implementation of a LHS may help foster a culture of learning and improvement for sustained success. Eight studies related to this finding and described how the LHS changed the culture within their healthcare organization [21, 23, 31, 33, 34, 39, 43, 45]. This included having a stronger culture of continuous learning and improvement, with several studies describing this feature as being crucial to LHS sustainability overtime. Sub-finidng 3b describes how health system leaders identify challenges in implementing LHSs, despite recognizing their value. Four studies aligned with finding 3b and reported the challenges of implementing a LHS [20, 24, 47, 50], such as facing financial barriers to implement and sustain the digital infrastructure [24] or in getting buy-in from key stakeholders [20]. Sub-finding 3c includes how LHSs may result in cost savings for the health system. Five studies talked about the potential cost savings of LHSs, following an economic analysis of their respective LHSs [28, 29, 31, 34, 35].

### Methodological quality

Of the 35 included studies, 12 were appraised for methodological quality [19–30]. The remaining 23 papers were grey literature or descriptive case studies and were therefore not eligible for appraisal. Studies were appraised using the relevant JBI appraisal tool and a score was assigned based on percentage of criterion met in each study. Quality scores ranged from 25 to 91%, with five studies receiving a score of 75% or higher [20, 21, 26–28]. Table 6 provides an overview of study scores for the 12 appraised studies.

**Table 6:** Overview of methodological quality for appraised studies (*n* = 12)

**Cohort studies**													
Author, Year	Q1	Q2	Q3	Q4	Q5	Q6	Q7	Q8	Q9	Q10	Q11	Score	%
Lowes, 2016	Y	Y	Y	Y	Y	Y	Y	Y	Y	U	Y	10/11	91
Mowry, 2020	N	N	Y	N	N	Y	Y	Y	Y	N	Y	6/11	55
**Qualitative studies**													
Author, Year	Q1	Q2	Q3	Q4	Q5	Q6	Q7	Q8	Q9	Q10	Score	%	
Dixon-Woods, 2020	N	Y	Y	Y	Y	Y	Y	Y	Y	Y	9/10	90	
Enticott, 2020	Y	U	Y	Y	Y	Y	Y	Y	Y	Y	9/10	90	
Jeffries, 2018	N	Y	Y	Y	Y	N	N	Y	Y	Y	7/10	70	
Nash, 2022	N	N	Y	Y	Y	N	N	Y	Y	Y	6/10	60	
Steels, 2021	N	N	Y	Y	Y	N	N	Y	Y	Y	6/10	60	
**Quasi-experimental studies**													
Author, Year	Q1	Q2	Q3	Q4	Q5	Q6	Q7	Q8	Q9	Score	%		
Vaughan, 2021	Y	U	U	N	Y	U	Y	Y	Y	5/9	56		
**Cross-sectional studies**													
Author, Year	Q1	Q2	Q3	Q4	Q5	Q6	Q7	Q8	Score	%			
Bhandari, 2016	Y	Y	Y	Y	N	N	Y	Y	6/8	75			
Floyd, 2019	N	N	U	Y	N	N	U	Y	2/8	25			
Miller, 2020	Y	Y	Y	Y	U	N	Y	Y	6/8	75			
Noritz, 2018	Y	Y	Y	Y	N	N	Y	U	5/8	63			

*N* no, *Qx* Question number *U* unsure, *Y* yes

## Discussion

Learning health systems offer a promising approach to advance VBHC; however, it is unclear how LHSs are operationalized across different health care organizations and countries. Our mixed methods review addresses this gap by highlighting and synthesizing the types of implementation strategies most used when transitioning to a LHS and provides some outcome data from functioning LHSs. Researchers and health system leaders may use these findings to support their own LHS implementation and evaluation efforts.

### LHS characteristics and implementation strategies

Studies highlighted the importance of digital infrastructure to capture data and integrate it back into the system to inform decision-making. This is a central component of LHSs described across the literature [6, 9, 54], and it is not surprising that this was a key finding in our review. Digital data capture was included as part of the LHS description in 31 studies, while digital infrastructure to support health services delivery was revealed as a key integrated finding across studies. Further, *changing record systems* was identified as the most common implementation strategy, highlighting that most health systems adapted their digital infrastructure in some way to implement their LHS. To support other LHS features, such as incorporating patient and provider experiences, and having timely production of evidence, it makes sense that establishing the appropriate digital infrastructure is a preliminary step. This aligns with previous LHS research that found a lack of infrastructure, digital registries and electronic systems for capturing patient data were common barriers to developing a LHS [10]. Clearly, establishing infrastructure for digital data capture is central to LHS implementation and therefore, researchers and health systems leaders may want to prioritize this aspect of implementation for advancing LHS transformation.

Only 21 studies included patient engagement as part of their LHS description, while one study included patient, consumer, and family feedback as an implementation strategy [36]. Another two studies reported involving patients or consumers to enhance the uptake and adherence of the LHS intervention [26, 42]. Evidence suggests that patient-centeredness in healthcare leads to improved patient outcomes and quality of care [55]. However, patient engagement has been lacking in LHS literature, with patient-clinician partnerships cited least often in a synthesis of LHS papers [10], and some LHS frameworks excluding this dimension altogether [54, 56]. While it is crucial not only to engage patients in LHS development, but to incorporate patient experience data back into the system, there are challenges in achieving this. Patients want LHSs to be transparent about the use of their data [57] and to have open communication and shared decision-making with their clinical provider within the LHS [58]. Patient-centred care should be central to any healthcare system, including LHSs. However, more research is needed to understand how to best engage patients in the development, implementation, and evaluation of LHSs to ensure health system transformation remains patient-centered.

Along with patient engagement, aligned governance and a culture of rapid learning and improvement were reported the least often (in 21 papers each). Only eight studies [35, 36, 41, 42, 44, 45, 47, 53] touched on all seven LHS characteristics in their study description. While it is possible that authors were not comprehensive in their LHS reporting, the findings from this review clearly indicate that key components of LHSs are not consistently reported across the literature. Aligned governance is a challenging feature to implement in LHSs [10, 59], largely due to ethical issues in policies and regulations [10, 60]. There are also barriers to establishing a culture of learning and improvement in LHSs due to low buy-in from health system stakeholders [35, 61]. This was a key integrated finding of this review, reported in multiple studies [20, 24, 47, 50]. Regardless, this aspect of the LHS was also recognized as important for ensuring it would be sustained overtime [43, 48, 49, 52]. Clearly, all LHS characteristics are important but there are challenges in incorporating each feature, which means some LHSs are missing key components. An evaluation framework or checklist would be useful to address these challenges in LHS implementation and to ensure researchers are meeting the requirements of a fully functioning LHS.

In this review, we used ERIC’s taxonomy of 73 implementation strategies to code how LHSs were implemented in health service organizations. Of the 73, only 33 distinct implementation strategies were employed, with changing record systems cited as the most common. Much of the literature highlights the need for a culture of rapid learning and improvement to facilitate LHS transformation [21, 49, 62]. Despite this critical component, few studies identified in our review employed implementation strategies that would adequately target culture change. While changing record systems are required to support the data linkage component of a LHS, LHSs will never be fully realized unless strategies aim to facilitate a culture of rapid learning and improvement. Future LHS initiatives should consider building on the strategies identified in this review including conducting local consensus discussions and building a coalition. Future LHS research should test additional implementation strategies that have not yet been evaluated for LHS implementation, including strategies that facilitate the development of stakeholder interrelationships and support clinicians to engage in LHS activities [63].

### LHS outcomes

Of the 35 studies in this review, 17 reported positive patient outcomes following implementation of their LHS. This finding is promising as one of the main goals of a LHS is to achieve VBHC, including improving patient outcomes and providing better quality care [2]. However, it is unclear what mechanisms directly led to improved patient outcomes and whether certain LHS features are more strongly associated with positive outcomes. Few studies reported on outcomes related to provider, population and health system costs and most studies did not evaluate the impact of LHS implementation. There is a need for further evaluation research to explore the full impact of LHSs, including how well it addresses the quadruple aim. Finally, the majority of LHSs in this review targeted a particular health condition or patient population. These LHSs were often conducted at an individual department level or as a multi-institutional network with several condition-specific departments working together. Few studies reported on a LHS at the organization level, such as across an entire hospital. This aligns with findings from a recent review that found only four of 76 studies described a LHS as an entire hospital system [64]. With the concept of LHSs still new, it makes sense that researchers may want to first establish a LHS for a particular patient population before expanding more broadly across an institution. However, there are examples of larger scale LHSs, such as the Swiss LHS, being implemented nationally across Switzerland [65]. It is important for researchers to learn from these broader health system examples to continue to scale and spread the efforts and impact of the LHS model.

### Implications for research, practice and policy

This comprehensive mixed methods systematic review illustrates important implications for research and health system leaders. First, this review highlighted the value of having a robust infrastructure to support digital data capture when implementing a LHS. Health system leaders and researchers should to prioritize this aspect of LHSs for an effective transition. Second, efforts are needed to support the engagement of patients into the development, implementation, and evaluation of LHS. Third, although many strategies are described in the implementation science literature, few were described in the included LHS studies. Future research should test additional strategies that facilitate partnerships development and engaging clinicians in LHS. Lastly, this review provides evidence that LHSs can lead to improved patient outcomes but there is a need for further evaluation studies on the overall effectiveness of LHSs and their impact on patient, provider, population and cost-related outcomes. With most LHSs being implemented at either an individual clinical unit or a multi-institutional network for a particular medical condition, there is a need for future implementation research to explore large scale health system transformation, such as organizational (e.g., entire hospital), provincial or state-wide health system transformation.

### Strengths and limitations

A key strength of this review is the comprehensive mixed methods approach. With the literature on LHSs continuing to emerge, it was important to capture the broad range of studies related to implementation. The inclusion of grey literature allowed for additional case study examples of LHSs to be explored and synthesized. Several aspects of implementation and LHS characteristics were used to categorize studies, which may be useful for health systems researchers and administrators to understand how they can apply similar approaches in their respective healthcare settings. A limitation of the current study includes the potential for biases in the critical appraisal process. While the studies were appraised by one reviewer and verified by a second, some of the questions in the JBI appraisal tool require interpretation of the authors, which allows for potential bias. Additionally, some of the studies included in this review did not have an appraisal tool that fit the methodology, and thus were unable to be appraised. The inconsistent and evolving LHS terminology was a challenge in this review, as some studies may have been excluded if they did not explicitly refer to their intervention as a LHS. This was necessary to avoid irrelevant papers but may have unfairly excluded studies that were truly LHSs but used lesser-known terminology. While no restrictions were placed on language or country, the majority of included studies were conducted in high-income, English-speaking countries. There is a need to explore LHSs in the context of low and middle-income countries.

## Conclusion

In this mixed methods systematic review, we described the implementation of LHSs in various healthcare settings, including implementation strategies, outcome measures, and components of functioning LHSs. As the field of LHS science and practice continues to advance, research is needed to better understand the impact of LHSs on patient, provider and population outcomes, and health system costs. Health systems researchers should continue to apply the LHS concept in practice, with a stronger focus on evaluating implementation strategies and outcomes.

### Supplementary Information

**Additional file 1: Table S1.** Example search strategy for online database, CINAHL (EBSCO), conducted July 28, 2022. **Table S2.** Detailed grey literature search strategy, conducted July 20, 2022. **Table S3. **JBI critical appraisal tool checklist questions. **Table S4. **Detailed overview of learning health system characteristics.

## Data Availability

All data generated or analyzed during this study are included in this published article and its supplementary information files.

## Abbreviations

AHRQ: Agency of Healthcare Research and Quality
ATN/AIR-P: Autism Treatment Network & Autism Intervention and Research Network on Physical Health
CHC: Connected Health Cities
CHOIR: Collaborative Health Outcomes Information Registry
CORE: Center for Outcomes Research and Evaluation
COVID: Coronavirus disease
EHR: Electronic Health Record
EQUIPPED: Enhancing Quality of Prescribing Practices for Older Adults in the Emergency Department
GERD: Gastroesophageal reflux disease
GHS: Geisinger Health System
HCA: Hospital Corporation of America
IBD: Inflammatory bowel disease
ICN: Improve Care Now
IDEA4PS: Institute for the Design of Environments Aligned for Patient Safety
LFEP: Learn from every patient
LHS: Learning health system
MS PATHS: Multiple Sclerosis Partners Advancing Technology and Health Solutions
MSQC: Michigan Surgical Quality Collaborative
NPCQIC: National Pediatric Cardiology Quality Improvement Collaborative
PCICCN: Post COVID-19 Interdisciplinary Clinical Care Network
OPQC: Ohio Perinatal Quality Collaborative
Peds-CHOIR: Pediatric adaptation of the Collaborative Health Outcomes Information Registry
QA: Quality Appraisal
RCLS-CF: Registry-enabled Care and Learning System for Cystic Fibrosis
SCK: Sickle cell knowledge base
SHOnet: Shriners Hospitals for Children (SHC) Health Outcomes Network
SPS: Solutions for Patient Safety
SNEPT: Starzl Network for Excellence in Pediatric Transplantation
UK: United Kingdom
USA: United States of America
VA-ESP: Veterans Affairs Evidence Synthesis Program
VBHC: Value-based healthcare
