# Supplementary material for: Implementation strategies and outcome measures for advancing learning health systems: a mixed methods systematic review
Source: Health Res Policy Syst. 2023 Nov 27;21:120. doi: 10.1186/s12961-023-01071-w (PMC10680228; doi:10.1186/s12961-023-01071-w)
Supplement: Supplementary file 1 — Additional file 1: Table S1. Example search strategy for online database, CINAHL (EBSCO), conducted July 28, 2022. Table S2. Detailed grey literature search strategy, conducted July 20, 2022. Table S3. JBI critical appraisal tool checklist questions. Table S4. Detailed overview of learning health system characteristics. [file 12961_2023_1071_MOESM1_ESM.docx]

Additional file 1 Tables

**Table S1.** Example search strategy for online database, CINAHL (EBSCO), conducted July 28, 2022

| 1 | (MH "Learning Health System") | 29 |
| --- | --- | --- |
| 2 | TI ("learning health system*" OR "learning health care system*" OR "learning healthcare system*" OR "rapid learning system*" ) OR AB ( "learning health system*" OR "learning health care system*" OR "learning healthcare system*" OR "rapid learning system*" ) | 520 |
| 3 | 1 or 2 | 527 |
| 4 | Limiters - Published Date: 20210101-20221231 | 112 |

**Table S2.** Detailed grey literature search strategy, conducted July 20, 2022

| **Date** | **Organization name & website URL** | **Search strategy(s)/ words searched including (if applicable) how items were selected*.*** | **# items retrieved/ search results** | **# of items screened** |
| --- | --- | --- | --- | --- |
| July 20, 2022 | <https://www.mcmasterforum.org/> | Used website’s search bar. Reviewed first 5 pages. Search terms:   1. Learning health system 2. Learning healthcare system 3. Rapid learning health system 4. Learning health systems and Implementation 5. Learning healthcare systems and Implementation 6. Learning health care systems and Implementation 7. Rapid learning health systems and Implementation 8. Rapid learning systems and Implementation | 1. 738 2. 428 3. 455 4. 478 5. 349 6. 445 7. 319 8. 319 | 1. 6 2. 0 3. 0 4. 1 5. 1 6. 1 7. 0 8. 0 |
| July 20, 2022 | <https://www.ahrq.gov/> | Used website’s advanced search bar. For searches with hundreds of returns, the first 5 pages were reviewed. Search terms:   1. Learning health systems and Implementation 2. Learning healthcare systems and Implementation 3. Learning health care systems and Implementation 4. Rapid learning health systems and Implementation 5. Rapid learning systems and Implementation 6. Learning health system 7. Learning healthcare system 8. Learning health care system 9. Rapid learning system | 1. 2 2. 0 3. 0 4. 1 5. 0 6. 395 7. 65 8. 54 9. 1 | 1. 0 2. 0 3. 0 4. 0 5. 0 6. 22 7. 3 8. 1 9. 0 |
| July 20, 2022 | <https://learninghealthcareproject.org/> | Used website’s tabs (no search function) to identify relevant articles. For searches with hundreds of returns, the first 5 pages were reviewed. Search terms:   1. <https://learninghealthcareproject.org/introduction-and-rationale/case-studies/> 2. <https://learninghealthcareproject.org/publications/> | 1. 18 case studies 2. 400 | 1. 4 2. 1 |
| July 20, 2022 | Advanced Google Search (google.ca) | Used advanced function to search for key terms. Reviewed first 5 pages of sources. Search terms:   1. Learning health system 2. Implement and learning health system 3. Learning healthcare system 4. Learning health care system 5. Rapid learning system 6. Learning healthcare system and implement 7. Learning health care system and implement 8. Rapid learning system and implement 9. Rapid learning health system | 1. 206, 000 2. 156,000 3. 85,000 4. 94,000 5. 39,000 6. 41,400 7. 86,000 8. 20,800 9. 7,680 | 1. 5 2. 4 3. 3 4. 1 5. 1 6. 3 7. 0 8. 2 9. 0 |

**Table S3. JBI critical appraisal tool checklist questions**

| **Study Design** | **Questions** |
| --- | --- |
| Qualitative | Q1: Is there congruity between the stated philosophical perspective and the research method  Q2: Is there congruity between the research methodology and the research question or objectives?  Q3: Is there congruity between the research methodology and the methods used to collect data?  Q4: Is there congruity between the research methodology and the representation and analysis of data?  Q5: Is there congruity between the research methodology and the interpretation of results?  Q6: Is there a statement locating the researcher culturally or theoretically?  Q7: Is the influence of the researcher on the research, and vice- versa, addressed?  Q8: Are participants, and their voices, adequately represented?  Q9: Is the research ethical according to current criteria or, for recent studies, and is there evidence of ethical approval by an appropriate body?  Q10: Do the conclusions drawn in the research report flow from the analysis, or interpretation, of the data? |
| Cross-sectional | Q1: Were the criteria for inclusion in the sample clearly defined?  Q2: Were the study subjects and the setting described in detail?  Q3: Was the exposure measured in a valid and reliable way?  Q4: Were objective, standard criteria used for measurement of the condition?  Q5: Were confounding factors identified?  Q6: Were strategies to deal with confounding factors stated?  Q7: Were the outcomes measured in a valid and reliable way?  Q8: Was appropriate statistical analysis used? |

Quasi-experimentalQ1: Is it clear in the study what is the ‘cause’ and what is the ‘effect’ (i.e., there is no confusion about which variable comes first)?

Q2: Were the participants included in any comparisons similar?

Q3: Were the participants included in any comparisons receiving similar treatment/care, other than the exposure or intervention of interest?

Q4: Was there a control group?

Q5: Were there multiple measurements of the outcome both pre and post the intervention/exposure?

Q6: Was follow up complete and if not, were differences between groups in terms of their follow up adequately described and analyzed?

Q7: Were the outcomes of participants included in any comparisons measured in the same way?

Q8: Were outcomes measured in a reliable way?

Q9: Was appropriate statistical analysis used?

**Table S4.** Detailed overview of learning health system characteristics

| **LHS Characteristic (Lavis et al.)** | **Examples from studies** |
| --- | --- |
| Patient engagement | **AHRQ (d) 2019:** patient experience data is collected through daily surveys which provide insight into the health system functions  **Bhandari 2016**: patients & caregivers complete the Peds-CHOIR survey before each appointment  **Britto 2018:** the NPC-QIC LHS includes a group of parents who lead the working groups and research committees  **Dixon-Woods 2020**: co-production with patients & clinicians is a key feature of the LHS  **Enticott 2020**: Involvement of consumers in LHSs was viewed as important and transparency around how patient data would be used was key to a sustainable LHS  **Forrest 2014**: PEDSnet’s purpose is to establish a community of patients, families, scientists, clinicians and health system leaders to work together in a LHS  **Fung-Kee-Fung 2018:** The Ottawa Health Transformation Model was developed by a community of practice, including patients  **Koscielniak, 2022:** SHOnet involves collaboration with system leaders, patients and families to support research and impact clinical practice  **Kreung, 2015:** patient data is entered by physicians from the electronic health record and by patients through self-reported data  **Kraphol, 2020:** patient experience and outcome data informed a postsurgical opioid prescribing program using the MSQC LHS model, with patient data used to generate new knowledge and inform practice  **Levin, 2022:** patient engagement occurred at the outset of the PC-ICCN development to ensure network was patient-oriented  **Mowry 2020**: Technology aimed to allow self-administered clinical assessment by patients  **Murray 2019**: Family councils are engaged in each step of the ATN/AIR-P LHS; Data from parents are integrated into the learning network to inform opportunities for improvement  **Nash, 2022(b):** clients, caregivers and community members are engaged throughout the learning cycle to determine their needs, experiences and interests  **Noritz, 2018:** clinical care team meets after each clinic to develop a care plan for each patient  **Perito 2021:** the patient and family voice council engage in ongoing meetings to build SNEPT  **Porcaro, 2022:** ICN uses a collaborative patient registry to inform research and quality improvement and includes multiple stakeholders, including patients and families, who provide input  **Satveit 2018**: MyAva was developed by understanding and incorporating patient experiences, involving a patient advisory board and targeted to patient needs  **Sinnige 2022:** ClaudicoNet includes informational videos for patients, which have also been developed based on practice observations  **Steels 2021:** Gathering patient and public views were critical elements of the CHC LHS, including having a designated role to enable patient voices to be heard  **The Learning Healthcare Project 2015:** patients are included as stakeholders who generate and use data within the system |
| Digital capture, linkage and timely sharing of relevant data | **AHRQ(a) 2019:** Baylor Scott & White Health is implementing a standardized data platform with a single EHR across all sites so that data can be accessed and shared more easily  **AHRQ(b): 2019:** Denver Health implemented a system wide EHR in 2016 & invested in infrastructure to support data use  **AHRQ (c): 2019:** HCA is using data to both address patient needs and to improve the health system more broadly  **AHRQ (d): 2019:** Utah Health includes a data warehouse that contains all the data ever collected in the health system, and is the heart of the LHS  **Bhandari 2016**: Peds-CHOIR collects patient data and tracks longitudinal outcomes across developmental phases to support care for patients with chronic pain from childhood to adulthood  **Britto 2018:** many learning networks include data registries that inform patient care, research and improvements  **Dixon-Woods 2020**: New forms of technology were implemented to enable data sharing. The dashboard allows for patient-clinician shared decision making and adjustments of treatment to optimize care  **Enticott 2020**: data linkage was viewed as important for producing valuable health care information and there should be better linkage of data between health care and other social care systems  **Forrest 2014**: ImproveCareNow implemented facilitated data entry from electronic health records; PEDSNet combined data from six institutions to create a data set with information on 1.4 million children  **Fung-Kee-Fung 2018**: Electronic health records help to identify care gaps from operational and clinical data  **Heys, 2022**: Neotree uses data from a digital application along with data visualization to support prenatal patient care, including incorporating audit and feedback data to support decision making  **Jeffries 2018:** Pharmacists used the dashboard to identify high-risk patients and adjusted their care using electronic health records  **Keung, 2015**: TRANSFoRm embeds clinical research into clinical practice through the electronic health record  **Koscielniak, 2022:** SHOnet was adapted from the PEDSnet digital infrastructure and uses data from the EHR  **Kraphol, 2020:** clinical outcome data from 70 hospital sites are centrally housed, with audit-and-feedback mechanisms in place to inform individual clinicians about their performance  **Levin, 2022:** a patient registry was created for systematic data collection  **Lowes 2017:** data were extracted from the electronic health record as well as billing data from the data warehouse  **Miller 2020**: A summary Smart Form was developed and integrated into the electronic health record to capture clinical data  **Mowry 2020**: Each participating center implemented a centralized health information database to transfer data  **Murray 2019**: Data is captured from parents through a web portal and/or application  **Nash, 2022 (b):** a common information system was developed for all organizations to collect and store data  **Noritz 2018:** Research data is incorporated into the electronic health record system that is driven by physician-inspired research questions to improve patient care  **Perito 2021**: Establish sources of data that will inform learning cycles and link to electronic health record data  **Porcaro 2021:** each member center contributes data to the ICN patient registry  **Satveit 2018**: a personalized dashboard allows both patients and providers to access data  **Sinnige, 2022:** a website and digital platform was created for therapists to use and collect routine data  **Steels, 2021:** a major challenge of the CHC LHS was the difficulty in accessing data and lack of electronic data sets  **Taylor, 2021:** outcome data were captured electronically through routine care  **The Learning Healthcare Project 2015:** Geisinger Health moved from analogue to digital data which allows for leveraging and using data in multiple ways  **Vandenberg, 2020:** linked data to an electronic health record to reduce the prescribing of potentially inappropriate medications  **Varnell 2022:** the EHR was altered to capture data and populate a dashboard with information |
| Timely production of research evidence | **AHRQ(a): 2019:** Baylor Scott & White Health have invested in research and analytics to incorporate learning and knowledge generation into daily activities  **AHRQ(b) 2019:** Denver Health is increasingly looking at ways in which data can be tracked and analyzed in real time  **AHRQ (c) 2019:** HCA uses natural language processing software which allows cancer coordinators to have more time to support patients, and patients receive test results more quickly  **Bhandari 2016**: Providers log into Peds-CHOIR to view results before and during an appointment with the family  **Britto 2018:** the registry includes access to data in real-time which is used to support clinical care, research and improvement activities  **Dixon-Woods 2020:** The LHS infrastructure includes a dashboard with patient data which can be accessed by both patients and clinicians to understand interactions and adjust care to optimize clinical management  **Enticott 2020**: timely access to relevant data was identified by participants as crucial for creating sustainable LHSs  **Floyd, 2019:** survey of LHS evidence synthesis users indicates evidence products are highly actionable and commonly used within 3 months of completion  **Forrest 2014**: ImproveCareNow reviews the entire population of patients each month to identify patients with missed care & share knowledge about how to implement changes and rapidly integrate new information into patient care  **Fung-Kee-Fung 2018**: performance dashboards with provincial metrics allow for timely corrective action  **Heys, 2022:** open data sets are used to share information throughout the country with policy makers and clinicians to improve neonatal health outcomes  **Jeffries, 2018:** pharmacists and physicians have daily access to a real-time surveillance dashboard which includes safety indicators for potentially hazardous prescribing  **Keung, 2015:** TRANSFoRm reuses routinely collected data from the EHR to pre-populate the case report form  **Koscielniak, 2022:** SHOnet continues to refine, expand, utilize and sustain the rapid generation of knowledge and translates that into care  **Kraphol, 2020:** patient data collected over several months was used to change prescribing practices among clinicians  **Levin, 2022:** PC-ICCN facilitates knowledge translation through a multimodal approach to dissemination; Knowledge was generated in a way to rapidly inform patient care  **Lowes, 2017:** the EHR uses patient data, such as missed appointments or new hospital admissions, to notify the care coordinator so that appropriate care can be planned and provided in a timely manner  **Miller, 2020:** patient data is entered into the Smart Form on an ongoing basis and is reviewed by team members at a weekly meeting; Analysis revealed high compliance among sickle cell disease providers for timely completion of patient data entry  **Murray, 2019:** data are continually generated and collected through the clinical process and are linked across learning network sites  **Nash, 2022 (b):** the Alliance is considering creating an ethics committee to enhance the research process  **Noritz, 2018:** the clinical team determined the data points to be collected during routine care that would provide evidence for clinical research and quality improvement work  **Perito, 2021:** collect and analyze data, identify areas for improvement, then implement and disseminate practice change based on evidence  **Satveit, 2018:** myAva aims to deliver timely care to women with the care team and scientific team delivering results to women immediately as it becomes available |
| Appropriate decision supports | **AHRQ(a) 2019:** Baylor Scott & White Health built a culture around using data to inform decisions so that staff are empowered to identify opportunities for improvement  **AHRQ(b) 2019:** Denver Health recognizes the importance of data and evidence-informed decisions to improve patient care, so investments in infrastructure focus on tools that allow providers to better help their patients  **AHRQ (c) 2019:** HCA includes a team of data scientists to apply machine learning, natural language processing and artificial intelligence to support how the health system improves patient care  **AHRQ (d) 2019:** Utah Health has invested in teams that analyze datasets and deliver information that can be used across the health system  **Bhandari, 2016:** the Peds-CHOIR database is maintained and administered with institutional technology support  **Britto 2018:** all networks include an actor-oriented network structure that allows them to self-coordinate and make decisions, solve problems and inform the health system  **Dixon-Woods, 2020:** data is shared between patients and clinicians during clinic visits to enable discussions, planning and shared decision-making, leading to better health outcomes for patients  **Enticott, 2020**: collaboration between data specialists, clinicians and academics, along with governance, systems and structures was seen as important to facilitate sustainable LHSs  **Floyd, 2019**: survey includes prompts for users including nature of decision-making needs, actions resulting from the report, implementation timeframe and perception of report content...only 6% of reports were not used and evidence synthesis reports were used to support intervention adoption decisions and medical device/therapy acquisition  **Forrest, 2014:** daily reports made available for each patient to improve care at the individual level with decision support and pre-visit planning for providers  **Fung-Kee-Fung, 2018:** patient priorities were incorporated in the design of integrated care pathways which influence change at the individual, departmental and operational levels  **Heys, 2022:** data is captured and used to inform clinical decisions at the bedside  **Jeffries, 2018:** the intervention allowed for a full review of patient medications so that the pharmacist could work with the physician to resolve any potential risks associated with hazardous prescribing  **Koscielniak, 2022:** a key aim of SHOnet is to use data and research to inform patient care with support from a fully functioning and sustainable data infrastructure  **Kraphol, 2020:** clinicians and institutions may use data from the MSQC system to inform quality improvement initiatives to improve practice and patient outcomes  **Levin, 2022:** the PC-ICCN patient registry supports clinical outcomes and health services research  **Lowes, 2017:** the objective of LFEP is to integrate clinical care and research and use this knowledge to deliver quality improvement in clinical care  **Miller, 2020:** the SmartForm populates relevant data for each patient by highlighting key biomarkers, comorbidities and treatments, which is then used by clinicians  **Mowry, 2020:** data from the patient-administered clinical assessment questionnaire is immediately available to health care providers through the electronic health record  **Murray, 2019:** leadership wanted to create a system for measuring change and to understand what is and isn’t working in order to accelerate improvements in care  **Nash, 2022 (b):** the dashboard uses data from the EMR to inform clinical decisions  **Noritz, 2018**: the data generated within the electronic health record system as part of the LHS, allowed clinicians to make evidence-based decisions around patient care  **Satveit, 2018:** the mAva platform uses multiple data sources to support clinical decision making among providers  **Sinnige, 2022:** ClaudicoNet ensured data collection efforts best inform real-time clinical decisions; Data is fed from the EHRs to therapists and then into the ClaudicoNet register to improve patient care  **The Learning Healthcare Project 2015:** the EHR collects patient data to inform how to guide the clinical consultation  **Vandenberg, 2020:** a key feature of EQUIPPED is to use clinical decision support to guide practitioners away from potentially inappropriate medications  **Varnell, 2022**: Guideline recommendations were integrated into the EHR to guide clinical decisions |
| Aligned governance, financial and delivery arrangements | **AHRQ(a): 2019:** Baylor Scott and White Health identified a need to implement structures that support innovation from both leaders down and frontlines up  **AHRQ(b) 2019:** Denver Health applies the concept of ‘Lean Kaizen’ in its culture, emphasizing that everyone in the system has a role to play in order to be successful  **AHRQ (c) 2019:** HCA put in place people to ensure the health system is set up to learn, iterate, disseminate knowledge and launch improvements across all sites  **AHRQ (d) 2019:** Utah Health restructured its approach to leadership which now allows for two-way communication about system and department-level priorities  **Britto 2018:** networks regularly evaluate their policies as part of their strategic planning and include membership policies, data sharing arrangements, research and regulatory policies  **Endicott, 2020:** participants reported transparent governance, systems and processes around LHS roles and responsibilities, and legislation around patient data sharing as crucial to sustainable LHSs  **Forrest, 2014:** the governance, regulatory, informatics, social and scientific infrastructure of PEDSnet will allow for research on multiple pediatric conditions  **Fung-Kee-Fung, 2018:** the regional community of practice united clinical, strategic leadership with operational and business management to improve care pathways  **Heys, 2022**: procedures are in place around data governance to ensure appropriate use of clinical data across health centres  **Koscielniak, 2022:** SHOnet aims to establish scientific and regulatory processes and procedures to guide data sharing, management and security  **Kraphol, 2020:** leadership should gather feedback from the collaborative membership and encourage frequent meetings and site visits to demonstrate the benefit of ongoing involvement to participants  **Levin, 2022:** the governance of PC-ICCN includes a core steering group, clinical care working group, a research group and a data information working group  **Moffat-Bruce, 2018:** stakeholder leaders had to commit to both financial and non-financial resources to make the transition to a LHS successful  **Mowry, 2020:** a steering committee was established, including representatives from health care institutions, industry partners and researchers, to guide decisions about the project  **Murray, 2019:** a design team including parents, clinicians, researchers and biostatisticians was established to guide the overall mission and goal of the Autism Learning Health Network  **Nash, 2022 (b):** a group of Alliance members work together to improve quality of care through continuous learning and collaboration  **Perito, 2021:** organizational structure established, decision-making processes were defined and trust-building was prioritized  **Porcaro 2021:** ICN was committed to stakeholder representation, with parents, patients and board members part of the network governance  **Satveit, 2018:** the medical advisory board guided the myAva team and supported physicians as they adopted the new technology in their practice  **Steels, 2021:** there was an agreement for data sharing and usage for each CHC site across the UK  **Taylor, 2021:** strong partnerships between academic, clinical and administrative leaders were key to LHS transformation  **Vandenberg, 2020:** All participating sites required buy-in from providers as the intervention targeted their behaviours |
| Culture of rapid learning and improvement | **AHRQ(a) 2019:** Baylor Scott & White Health has built a culture of learning and improvement through data, which empowers staff to implement changes and improve outcomes  **AHRQ(b) 2019:** Denver Health fosters a culture of learning where staff are encouraged to speak up and offer ideas for improving care  **AHRQ (c) 2019:** At HCA, using data in a continuous cycle of learning is part of its culture  **AHRQ (d) 2019:** Culture change started a decade ago at Utah Health and includes two key initiatives to support a culture of learning  **Britto 2018:** the networks established a culture of learning, with leaders promoting a shared responsibility for outcomes and facilitate learning from data  **Dixon-Woods, 2020:** the LHS aims to learn from the experiences and successes of other programs in order to improve patient care  **Enticott, 2020:** culture and processes for routinely integrating data into practice were seen as key to LHS sustainability  **Forrest, 2014:** PEDSnet aims to create a community of patients, families, clinicians, scientists and health care system leaders who work together in a LHS dedicated to ensuring the best care is delivered  **Fung-Kee-Fung, 2018:** a regional community of practice in lung cancer was established to engage multiple and diverse stakeholders from various institutions in the LHS redesign; Within this group, key change leaders united stakeholders and improved care processes  **Heys, 2022:** strong leadership, government buy-in, collaboration and stakeholder engagement have been key to the successful implementation of Neotree  **Koscielniak, 2022:** in Phase 2, SHOnet will expand the digital infrastructure to support learning and establish processes for conducting research and supporting a LHS culture  **Kraphol, 2020:** continuous, informative updates keep members informed and nurture the community of practice and comradery among members  **Levin, 2022:** collaboration between patients, clinicians, administrators, policy-makers and researchers allows for the dissemination and mobilization of new knowledge  **Moffat-Bruce, 2018:** a learning culture was established by reframing the role of research in improving outcomes and by including all stakeholders as equal partners and embedding them in the communities where research would take place  **Mowry, 2020:** initial collaborators realized the importance of building trust when establishing a LHS with an academic-industry partnership, so all parties aligned their vision to have a shared focus on generating evidence to improve patient outcomes  **Murray, 2019:** the first step in transitioning to a learning network was to obtain buy-in from stakeholders through frequent communication and education around the value of a LHS approach  **Nash, 2022 (b):** Alliance members and committees ensure all members, including clinicians, leaders and staff, are involved in decision making and work together to facilitate change  **Perito, 2021:** built a community of working groups with a culture of collaboration, transparency, innovation and continuous learning  **Satveit, 2018:** the medical advisory board members are excited by the myAva technology and are motivated to improve women’s health care  **Sinnige, 2022:** ClaudicoNet is run by a team of people with a range of knowledge and expertise who work together and exchange knowledge  **Steels, 2021:** there were challenges in establishing a culture of learning and improvement with a lack of consistent commitment to the project from all partners and noted differences between academic and industry working styles  **Taylor, 2021:** creating a learning culture requires a paradigm shift at both the macro and micro level with all stakeholders having common LHS goals and leaders who foster a culture of learning |
| Competencies for rapid learning and improvement | **AHRQ(a) 2019:** Baylor Scott and White Health built a workforce of people with data analysis and research skills to incorporate knowledge in system operations  **AHRQ(b) 2019:** Denver Health is rooted in a culture of continuous improvement with opportunities for leaders to gain experience and build relationships across different departments  **AHRQ (c) 2019:** HCA hosts learning summits to share successes and resources across sites and teams  **AHRQ (d) 2019:** the collaborative structure of Utah Health allows for information sharing between different stakeholders and departments, which lead to rapid improvements  **Britto 2018:** networks such as ICN, promote a culture of collaboration where everyone shares ideas and are engaged in teaching and learning from one another  **Bhandari, 2016:** follow-up evaluations indicated low adherence to Peds-CHOIR, prompting additional staff training, clinician education and discussions with patients, clinicians and staff about the benefits of Peds-CHOIR  **Dixon-Woods, 2020:** participants hoped that using co-production to determine treatment protocols would reduce the burden of data reporting and the opportunity for entering inaccurate data; Further interview data suggests that the benefits of the LHS are viewed more favorably than the potential concerns with surveillance  **Enticott, 2020**: a skilled workforce, capability and capacity for learning and using data to inform practice change were important in a LHS  **Forrest, 2014**: ImproveCareNow uses standardized processes such as reviewing population data and learning from changes in performance so that care centres can rapidly integrate new information into patient care. This allows for rapid responses to clinical questions that cannot be determined through clinical trials  **Fung-Kee-Fung, 2018:** the application of process improvement tools allowed for clinical learning opportunities for providers  **Heys, 2022:** with high staff turnover, adaptations were made to build simple and comprehensive educational features within Neotree; A paid Neotree ambassador was hired to provide technical support for Neotree during the implementation phase  **Jeffries, 2018:** pharmacists received training on the research topic, including safety indicators, root cause analysis and use of the dashboard prior to study commencement  **Koscielniak, 2022:** SHOnet supports a culture of learning and improvement through its infrastructure and approach to knowledge translation  **Kraphol, 2020:** partnerships between health care institutions allow for leveraging data and accelerating the adoption of evidence to practice by harnessing knowledge from diverse teams and learning from one another rather than reinventing solutions  **Levin, 2022:** a goal of PC-ICCN was to normalize the integration of research into clinical care and to create a culture of collaboration across different teams  **Miller 2020:** the SmartForm was built through multiple iterative validation cycles, with ongoing, continuous input from clinicians on how to enhance the tool  **Moffat-Bruce, 2018:** there is an institutional commitment to change practice regardless of whether a project receives funding  **Mowry, 2020:** MS Paths is enabled by technological infrastructure to support data transferring, processing and harmonization, with LHS data available upon request for research purposes by any participating health care institution  **Murray, 2019:** the shared vision of the Autism Learning Health Network allowed for a system for gathering evidence, implementing change and disseminating findings, with ongoing opportunities for formal training in quality improvement across participating networks  **Nash, 2022 (b):** members are encouraged to work with peers to foster a culture of learning in the workplace  **Perito, 2021:** provide training on LHS principles to families and providers  **Satveit, 2018:** providers are supported with the tools and intelligence to create treatment plans for their patients by using their expertise along with the data  **Sinnige, 2022:** ClaudicoNet is continuously improved through collaboration among a diverse group of stakeholders including therapists, patients, professional bodies and web-development companies  **Steels, 2021:** the CHC program had to develop an LHS infrastructure and working culture across multiple organizations and stakeholders  **Taylor, 2021:** a multidisciplinary team was established to support the scale up of the LHS across the health system, who facilitate ongoing training, mentorship and data efficiencies  **Vandenberg, 2020:** order sets were integrated in the clinical workflow and with continuous implementation efforts, sites increased visibility of order sets to providers |
